# International Opinion: The True Cost of Wounds for Canadians: 2025 Update

**DOI:** 10.1111/iwj.71096

**Published:** 2026-11-03

**Authors:** Douglas Queen, Mariam Botros, Keith Harding

**Affiliations:** ^1^ Medicalhelplines.com Inc Toronto Ontario Canada; ^2^ Wounds Canada Toronto Ontario Canada; ^3^ Cardiff University Cardiff UK

## 1 Introduction

In our previous editorial [1], we described how difficult it is to obtain consistent, comparable estimates of wound care costs across Canada. Provincial and national figures were available, but many were dated, based on different methods or difficult to compare. We therefore proposed a reproducible approach that combines publicly available government data on healthcare spending and population with internationally validated estimates of the share of health expenditure attributable to wound care.

The 2025 update uses the same method with current population and per capita healthcare spending data. The resulting estimates point to a marked rise in wound care expenditure, driven by population growth, ageing, chronic disease and higher per capita health costs. The authors have updated their original 2023 report annually in Wounds Care Canada [2, 3] but feel it is important to update our original report. In addition to updated cost estimates we will use a similar approach to estimate the numbers of wounds and their aetiological split, by geography. This provides additional information for wound care practitioners to present and discuss with policy makers and funders.

## 2 Methods

### 2.1 Cost Estimation Methodology

We estimated wound care expenditure using the proportional method described in our first cost‐based editorial by Queen and Harding [4]. International health economics studies generally place wound care costs at 2%–5% of total regional healthcare spending. The exact share varies with the mix of wound types, care settings, local service capacity and the quality of available data [5, 6, 7, 8, 9, 10, 11].

Because wound care services and data systems differ across jurisdictions, we used the median international estimate of 3.5% for every Canadian province and territory, consistent with earlier national updates [2, 3]. This conservative midpoint provides a defensible national estimate and allows year‐to‐year comparisons.

$$\begin{array}{cc} \text{Formula}:\text{Wound Care Spend}= & \;\text{Population}\\ & \;\times \mathrm{Per}\;\text{Capita Health Spend}\;\left(\mathrm{CAD}\text{\$}\right)\\ & \;\times 0.035. \end{array}$$

### 2.2 Wound Count Estimation

We estimated the annual number of wounds managed in Canada using the Adjusted Wound Prevalence Proportion (AWPP), following the approach of Queen and Botros [3].

Published estimates place wound prevalence at 1.5%–5% of the general population, depending on case definitions, care settings and the completeness of reporting [12, 13, 14]. We applied a conservative midpoint of 3%.

Formula:Number of Wounds=Population×0.03

To show how the estimated wound burden may be distributed by cause, we divided each provinces and territory's total across the major wound categories reported in the literature. Pressure injuries (17.5%) [15], leg ulcers (45%) [16] and diabetic foot ulcers (35%) [17] were calculated using the published proportion for each wound type. The balance was classified as ‘other wounds’, including surgical, traumatic, arterial, malignant and less common wounds (2.5%) [18].

$$\begin{array}{c} \text{Formula}:\text{Aetiological Estimates based}\;\mathrm{on}\;\\ \text{total wound count with the following attributions}-\mathrm{PI}\\ =\mathrm{WC}\times 0.175;\mathrm{LU}=\mathrm{WC}\times 0.45;\mathrm{DFU}=\mathrm{WC}\times 0.35\;\\ \text{and Other}=\mathrm{WC}\times 0.025. \end{array}$$

This method offers a transparent view of the likely mix of wound types nationally and within each province or territory. Canada does not have a comprehensive national wound registry or routinely reported surveillance data by wound aetiology, although some pressure injury reports are available in certain provinces. The figures presented are therefore modelled estimates rather than observed counts. They should be read as an indication of distribution based on published proportions applied to the overall estimated wound total.

### 2.3 Limitations

These estimates are based on published government data and internationally validated proportions; they are not drawn from a national registry or primary surveillance system, neither of which currently exists at sufficient scale in Canada, or in most geographies around the world. The latest systematic national wound data published by [spell this out] CIHI come from the 2011–2012 Continuing Care Reporting System cycle [19], leaving a gap of more than 13 years. The results are therefore best treated as order‐of‐magnitude estimates for policy discussion and resource planning, not as precise epidemiological measurements. Linking electronic health records with homecare and long‐term‐care reporting would substantially improve future estimates.

## 3 Results

### 3.1 National Overview

Canada's estimated wound‐care expenditure exceeded CAD $14 billion in 2025 for the first time ($14 007 230 342). The estimate has risen steadily from $11.0 billion in 2019 [1] to $12.1 billion in 2023 [2], $13.1 billion in 2024 [3] and $14 billion in 2025, an increase of about 27% over 6 years, or a compound annual growth rate of 4.1%. An estimated 1 247 268 wounds are managed in Canada each year.

### 3.2 Provincial and Territorial Cost Breakdown

Table 1 shows estimated wound‐care spending for all 13 provinces and territories. Ontario has the highest total at $5.1 billion, or roughly 36% of the national estimate.

**TABLE 1:** Estimated costs of wound care within Canada by province and territory (2025).

Province/territory	Population [20] (2025)	Per capita health [21] 2025 (CAD$)	Estimated total healthcare spend 2025 (CAD$)	Estimated wound care spend 2025 (CAD$)
Ontario	16 191 372	$8405	$146 450 959 740	$5 125 783 591
Quebec	9 058 089	$8984	$84 421 389 480	$2 954 748 632
British Columbia	5 683 201	$10 439	$59 326 935 239	$2 076 442 733
Alberta	5 040 871	$9597	$48 377 238 987	$1 693 203 365
Manitoba	1 507 057	$10 868	$16 378 695 476	$573 254 342
Saskatchewan	1 266 234	$10 191	$12 904 190 694	$451 646 674
Nova Scotia	1 091 857	$11 617	$12 684 102 769	$443 943 597
New Brunswick	868 630	$9431	$8 192 049 530	$286 721 734
Newfoundland & Labrador	549 738	$11 109	$6 107 039 442	$213 746 380
Prince Edward Island	182 508	$10 470	$1 910 858 760	$66 880 057
Yukon	48 261	$20 124	$971 204 364	$33 992 153
Northwest Territories	45 848	$28 714	$1 316 479 472	$46 076 782
Nunavut	41 919	$30 086	$1 261 175 034	$44 141 126
Canada (total)	**41 575 585**	$9626	**$400 206 581 210**	**$14 007 230 342**

Quebec follows at an estimated $2.9 billion, with British Columbia at $2.1 billion. Together, the three provinces account for more than 73% of national wound‐care expenditure, broadly in line with their combined share of Canada's population.

### 3.3 Provincial and Territorial Wound Count

Table 2 presents the estimated number of wounds managed annually by province and territory. Ontario accounts for the largest wound volume with an estimated 485 714 wounds annually, followed by Quebec (271 743) and British Columbia (170 496). At the territorial level, wound counts are generally small in absolute terms, approximately 1448 (Yukon), 1375 (Northwest Territories) and 1258 (Nunavut), yet these populations face among the most significant barriers to timely, evidence‐based wound care of any group in Canada [22].

**TABLE 2:** Estimated number of wounds within Canada by province and territory (2025).

Estimated total wounds and distribution by wound type					
Province or territory	Estimated wounds	Pressure injuries	Leg ulcers	Diabetic foot ulcers	Other wounds
Ontario	485 741	85 005	218 584	170 009	12 144
Quebec	271 743	47 555	122 284	95 110	6794
British Columbia	170 496	29 837	76 723	59 674	4262
Alberta	151 226	26 465	68 052	52 929	3781
Manitoba	45 212	7912	20 345	15 824	1130
Saskatchewan	37 987	6648	17 094	13 295	950
Nova Scotia	32 756	5732	14 740	11 465	819
New Brunswick	26 059	4560	11 727	9121	651
Newfoundland & Labrador	16 492	2886	7421	5772	412
Prince Edward Island	5475	958	2464	1916	164
Yukon	1448	253	652	507	43
Northwest Territories	1375	241	619	481	41
Nunavut	1258	220	566	440	38
Canada	**1 247 268**	**218 272**	**561 270**	**436 544**	**31 182**

*Note:* Values are estimates and are displayed to the nearest whole wound. Estimated wounds = 3.0% of population. Wound types are allocated as 17.5% pressure injuries, 45% leg ulcers, 35% diabetic foot ulcers and 2.5% other wounds.

Applying the published aetiology specific proportions to the derived wound counts indicates that leg ulcers represent the largest estimated wound category nationally, accounting for approximately 561 270 wounds. Diabetic foot ulcers represent the second‐largest category, with an estimated 436 544 wounds, followed by pressure injuries at approximately 218 272 wounds. The residual ‘other wounds’ category accounts for approximately 31 182 wounds nationally. This pattern was consistent across provinces and territories because the same proportional allocation was applied to each jurisdiction's total estimated wound count. These findings are best interpreted as an indicative national and provincial distribution of wound aetiology within the overall modelled wound burden, rather than as directly observed surveillance counts by wound type.

### 3.4 Wound Registry

Wound registries are already in use in the United Kingdom [23], Australia [24], Singapore [25] and the United States [26]. They provide evidence that supports research, clinical practice, policy and innovation.

Canada's lack of a national wound registry or real‐time surveillance system remains a major barrier to sound policy. As mentioned previously, the latest comprehensive CIHI data [19], was more than 13 years out of date. Since then, the population has grown by millions, diabetes has become more prevalent, and wound care products and services have changed substantially. Yet decisions about resources, models of care, workforce needs and performance are still being made without current national data on wound prevalence, incidence or outcomes. The rising costs reported here strengthen the case for a federally supported national wound registry.

## 4 Conclusions

The 2025 estimate puts Canada's annual wound care bill above $14 billion. That is 27% higher than 6 years ago. The increase reflects sustained pressures, population ageing, chronic disease and rising healthcare costs rather than a 1‐year anomaly. Wound care now warrants a national response that matches the scale of the burden.

The estimated 1 247 268 wounds managed each year also show where much of the burden lies. Leg ulcers, diabetic foot ulcers and pressure injuries are the largest modelled groups. Policy must therefore address not only total spending, but also the clinical pathways that shape wound development, recurrence, chronicity and preventable complications.

The clearest structural weakness is the absence of a national wound registry. A federally supported registry would give clinicians and policymakers the timely evidence they need and the cost trend documented in this series makes a strong fiscal case for that investment.

Wounds should not be accepted as an unavoidable cost of any modern health system. Even as estimates, these figures make the scale of the problem clear and point to the need for a coordinated national response.

## Conflicts of Interest

The authors declare no conflicts of interest.

## Data Availability

The data that support the findings of this study are available from the corresponding author upon reasonable request.
